# Three-dimensional multi-source localization of underwater objects using convolutional neural networks for artificial lateral lines

**DOI:** 10.1098/rsif.2019.0616

**Published:** 2020-01-22

**Authors:** Ben J. Wolf, Jos van de Wolfshaar, Sietse M. van Netten

**Affiliations:** Bernoulli Institute of Mathematics, Computer Science and Artificial Intelligence, Faculty of Science and Engineering, University of Groningen, Groningen, The Netherlands

**Keywords:** convolutional neural network, hydrodynamic imaging, inverse problem, lateral line, sensor array, source localization

## Abstract

This research focuses on the signal processing required for a sensory system that can simultaneously localize multiple moving underwater objects in a three-dimensional (3D) volume by simulating the hydrodynamic flow caused by these objects. We propose a method for localization in a simulated setting based on an established hydrodynamic theory founded in fish lateral line organ research. Fish neurally concatenate the information of multiple sensors to localize sources. Similarly, we use the sampled fluid velocity via two parallel lateral lines to perform source localization in three dimensions in two steps. Using a convolutional neural network, we first estimate a two-dimensional image of the probability of a present source. Then we determine the position of each source, via an automated iterative 3D-aware algorithm. We study various neural network architectural designs and different ways of presenting the input to the neural network; multi-level amplified inputs and merged convolutional streams are shown to improve the imaging performance. Results show that the combined system can exhibit adequate 3D localization of multiple sources.

## 1. Introduction

Fish are able to accurately sense nearby moving or vibrating objects in water via a lateral line organ [1]. This organ consists of superficial neuromasts (SNs) and canal neuromasts (CNs). The SNs are located externally on the skin and detect the outside flow velocity, whereas the CNs are located underneath the skin in small canals, which filter out low frequencies [2]. As a result, the CNs are sensitive to external flow acceleration in directions parallel to the canals of the organ. Fish are able to use the mechanosensory input elicited by these neuromasts for a variety of tasks, including tracking down prey [3] or coordinating their movements, e.g. in schooling [4].

This biological ability has inspired the development of flow-sensing arrays called artificial lateral lines (ALLs) to perform what is known as hydrodynamic imaging: mapping objects and obstacles via fluid flow interactions. Several ALL set-ups are used for wake detection [5] and source localization [6–8], usually benchmarked by their ability to localize a vibrating dipole source. Most ALL sensor configurations are focused towards a two-dimensional (2D) localization problem, while some use out-of-line sensor placement and template-matching methods to localize a phase-locked vibrating source in three dimensions [9].

The present research contributes to the development of algorithms for ALL hydrodynamic imaging systems as it is the first of its kind to consider localizing multiple objects in three dimensions using machine learning. The field of machine learning might provide the means to tackle these problems, since the resulting systems have exhibited impressive sensory-processing capabilities in a wide range of tasks that require a high degree of generalization, such as computer vision [10] and speech recognition [11].

To feasibly explore a number of different configurations of the artificial neural network, we consider a simulated environment in which objects in motion generate a velocity potential field, which is measured as fluid flow by the ALL. The underlying inviscid hydrodynamic flow model for the local velocity potential is supported by experimental findings in fish lateral line research [12–14]. In practice, this model may be compromised to create an adequate image of the near surroundings since it neglects vorticity and other possible issues that arise in reality. This demands a system that can cope with a highly dynamic environment and, from the point of view of supervised learning, should generalize well to unseen situations.

In this research, we apply machine learning to explore the applicability of ALL hydrodynamic imaging systems to create local images of moving objects by using simulated ALL inputs. We define the problem as the prediction of a probability function that can be related to the likelihood of a sphere to be in a certain position. This 2D probability function is then used to produce an estimate of the three-dimensional (3D) location using an iterative localization algorithm.

We test several configurations of a convolutional neural network (CNN) to determine the influence of several design choices. While one-dimensional (1D) inputs with CNNs have been used before [11,15], these applications perform dimensionality reduction. In our case, we use the CNN to transform a 1D signal to a 2D probability grid, which requires alterations to a standard CNN architecture. This dimensionality upscaling and specifically its application to the ALL is completely novel.

In §2, we discuss the hydrodynamic model and related research that is relevant to our approach. Section 3 elaborates on the details of the 3D source location-encoding model and the general CNN architecture. In §3.4 we discuss the comparative experiments that were conducted to measure the accuracy of our models for locating multiple sources and report the results in §4. Finally, we discuss our findings and list directions for future research in §5 and conclude in §6.

## 2. Background

First, we discuss other research on source location encoding with a lateral line organ. Then, we elaborate on the research and insights considering CNNs that inspired the development of our method.

### 2.1. Source location encoding by the lateral line organ

Source localization involves arrays of multiple sensors that measure a projection of the local fluid velocity potential in response to an object moving relative to the array.

**Figure 1.**
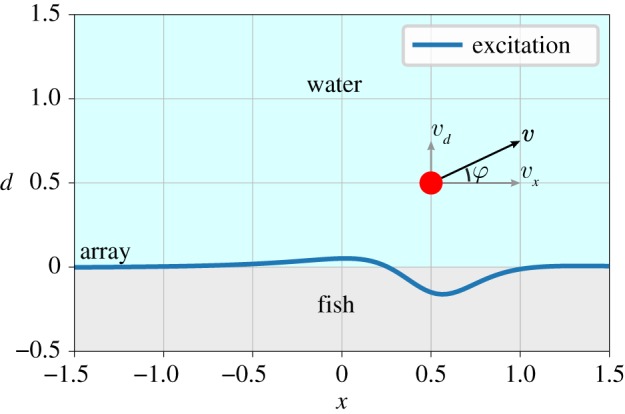
Illustration of a situation with a single sphere in a plane with the excitation pattern of a dense neuromast array at *d* = 0. (Online version in colour.)

Findings by Münz [16] and Bleckmann *et al.* [17] show that the hair cells in each neuromast sensor are aligned in one direction. This causes these sensors to effectively measure a 1D projection of the local pressure gradient as caused by, for instance, a moving object. When these objects are located in the vicinity of the array and they are sufficiently large, the effects of viscosity can be neglected [18]. These findings were used in [12] to develop a theoretical model and method to compute the pressure gradients between two lateral line canal pores. This model describes the neural excitation of neuromasts along the lateral line, also known as an excitation pattern. Figure 1 illustrates the resulting excitation pattern along a dense neuromast array as generated by a moving sphere.

In [12,13], it was demonstrated that the information about the location of a vibrating source is encoded in the spatial characteristics of the excitation pattern along the lateral line sensors. More specifically, Ćurčić-Blake & van Netten [12] demonstrate that the excitation of the sensors along the array can be determined from a combination of two wavelets

2.1 $$\psi_{\mathrm{even}}(s,x,d)=\frac{1-2({\left(s-x)/d\right)}^{2}}{{\left[1+({\left(s-x)/d\right)}^{2}\right]}^{5/2}}$$

and

2.2 $$\psi_{\mathrm{odd}}(s,x,d)=\frac{-3((s-x)/d)}{{\left[1+({\left(s-x)/d\right)}^{2}\right]}^{5/2}},$$

where *s* is the position of the neuromast along the *x*-axis and (*x*, *d*) is the position of the sphere. Here, *x* denotes the source position relative to the array and *d* is its distance. The actual fluid velocity measured by a neuromast as produced by a moving sphere can be obtained through

2.3 $$v(s,x,d)=\frac{{Wr}^{3}}{2d^{3}}(\psi_{\mathrm{odd}}\sin\varphi -\psi_{\mathrm{even}}\cos\varphi ),$$

where *W* is the sphere’s absolute velocity, *r* is its radius and φ is its direction with respect to the array. Note that the left factor of the right-hand side of this equation causes the fluid velocity to scale in a nonlinear way with respect to the distance (*d*) to the array.

### 2.2. Object localization using artificial lateral lines

Traditionally, template-matching methods have been used to create a 2D heat map [6] and in some cases a 3D volume [9] of a single source. More recently, artificial neural networks have been used for single source localization using both physical [19,20] and simulated [21,22] sensor arrays.

#### 2.2.1. Two-dimensional localization

For 2D localization of a single sphere, a classical lateral line geometry is often used, where all sensors are positioned equidistantly on a single line. Using the inviscid hydrodynamic model described in the previous section, one can readily determine the velocity potential at each of the sensors in this ALL from the relative location and motion of a source *S*^(*i*)^: the forward problem,

2.4 $$F(S^{\left(i\right)})=u_{\mathrm{obs}}.$$

The concatenation of these locally measured fluid velocities *u*_obs_ at each sensor is known as a velocity pattern, analogous to the excitation pattern. The effective problem statement that ALL set-ups face is the inverse problem; from the measured velocity pattern, reconstruct the relative position and motion of a source,

2.5 $$F^{-1}(u_{\mathrm{obs}})=S^{\left(i\right)}.$$

This inverse problem (*F*^−1^) has been tackled via template-matching and beamforming methods [6]. These methods use a library of modelled or measured velocity patterns and a correlation scheme to retrieve the source location of a newly presented velocity pattern.

An example of Capon’s beamforming is described in detail in [23]. Here, the authors make use of an outer-product correlation matrix between the sensors for a single time step. The second key component is a vast 3D library (*x*, *d*, φ) of modelled velocity patterns for a range of possible source locations and orientations. This matrix and library are then used to create a heat map with Capon’s method. The predicted location is finally determined by finding the maximum in this heat map.

In addition to these beamforming methods, non-convolutional neural networks have been used for 2D localization. A multi-layer perceptron (MLP) with one hidden layer having 24 nodes was applied to an ALL array consisting of six sensors in a row [19,20]. Here, the position of a vibrating sphere was varied and reconstructed in a 2D plane. In [21], the localization performance for a single source in a 2D plane was assessed for three different types of neural networks: the MLP, an echo state network, and an extreme learning machine (ELM); the last proved to be optimal. This type of network was also used in [14] to localize both moving and stationary vibrating sources in a 2D plane. Recently [24], the ELM architecture was compared with a recurrent network architecture (LSTM) for objects moving in a straight line in a 2D plane. To the authors' knowledge, the present work is the first effective demonstration of a CNN architecture for localizing a source with an ALL.

#### 2.2.2. Three-dimensional localization

The problem space around the classic ALL geometry is circle symmetric; one cannot discriminate distance in the *y* plane versus distance in the *z* plane. Other sensor geometries that break this symmetry are required to extend this problem to 3D localization.

In [9], a cross geometry was introduced on a cylinder, where nine 1D-sensitive sensors were positioned in a straight line with three sensors perpendicular at either side of the centre of the array. To localize the source in a 3D volume around the lateral line set-up, they extended the beamforming algorithm [23] to work with a five-dimensional library containing the (*x*, *y*, *z*) location and orientation (azimuth *θ* and zenith *ϕ* angles). As in [23], the location of the source was selected from the maximum in the 3D heat map volume. A slightly different approach is needed for localizing multiple sources.

In [25], two sources are positioned in a simulated 3D basin with several geometries of 16 sensors placed at the bottom of this basin. There, an artificial neural network was tasked to reconstruct a 3D heat map volume using the sampled fluid velocity at each sensor’s location. Yet, because the chosen geometries were spatially uncorrelated, the network could not make use of the spatial properties encoded in velocity patterns as sampled in a line.

Our chosen sensor geometry of two ALLs with equidistant sensors retains the ability to make use of the spatial properties [12,13], while allowing 3D localization. These spatial properties make this problem well suited to be learned by CNNs.

### 2.3. Convolutional neural networks

CNNs have received increasing attention in machine learning research in recent years. These networks are especially suited for 2D data-driven tasks for images such as the ImageNet Large-Scale Visual Recognition Competition (ILSVRC) in image classification, detection and visual segmentation tasks [10,26–28]. Other than computer vision, CNNs have also been applied to 1D signals in speech recognition [11] and haptic tactile classification [15].

CNNs differ from the more standard MLPs [29] in their more efficient architectural design and were initially proposed in their current form by LeCun *et al.* [30]. A key insight behind the development of this kind of network architecture is that the pixels in an image are spatially correlated. This means that meaningful features of an image, such as edges, corners and colour transitions, can be found in small subregions. This allows small 2D filters to be tuned for detecting these meaningful features. These filters can be tuned more easily, since they are trained using the larger collection of subregions rather than the whole image.

A CNN is usually made up of several convolutional layers, interlaced with pooling layers to reduce the dimensions of the convolutional stream. Typically, additional convolutional layers converge to filters that hierarchically extract more abstract features [31]. In the extreme case, an image can be reduced via several convolutional and pooling layers to a single output neuron for binary classification.

Our alterations to the standard implementation of the CNN are further described in §3.3.

## 3. Methods

**Figure 2.**
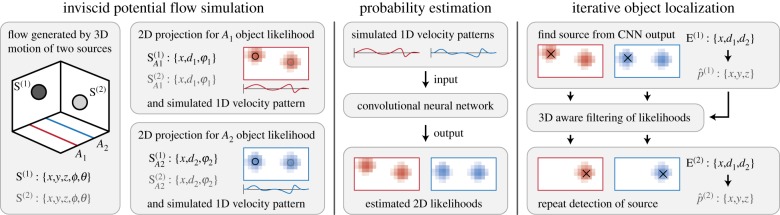
Schematic of the task and processing pipeline. The simulation generates examples of objects in motion, accompanied by a 2D projection of object probability and the 1D velocity pattern at each array. The overall task is to localize these objects using the 1D velocity patterns as input in two steps: estimating the 2D probability grids of a source present and then determining the coordinates of the object in three dimensions using these grids. (Online version in colour.)

This section elaborates on source location encoding in three dimensions, the exact simulation implementation, the global architecture of the CNN used and its tested variations, and the algorithm for iterative location decoding in three dimensions. An overview of the whole process is shown in figure 2.

### 3.1. Location encoding in three dimensions

We first consider the situation of a single sphere in a 2D plane, after which we extend it to multiple spheres and a 3D volume.

#### 3.1.1. Spheres in a plane

With a single sphere in a plane, the object motion is described by a speed *W* and direction φ. When a sensor array is positioned at *d* = 0, we can determine the fluid velocity at each sensor *s* using the source’s position (*x*, *d*) via the fluid flow model as described by equation (2.3).

**Figure 3.**
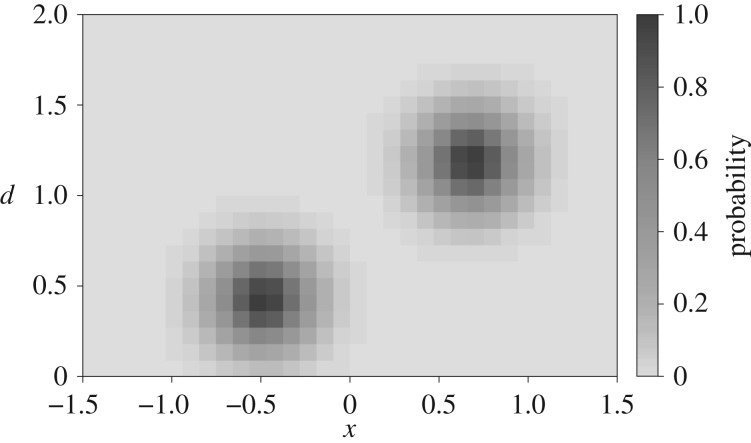
Plot of the target probability function in a 2D grid for two sources. The target function is evaluated at (*x*, *d*), where *x* is taken from 32 equidistant points between −1.5 and 1.5 and *d* is taken from 22 equidistant points between 0 and 2.0.

With the location of the source encoded in the velocity pattern, we can localize the source by effectively decoding this profile. If only a single sphere is considered, localization can be treated as a regression problem in which an inverse method predicts the components of a position vector p. However, if the number of spheres is arbitrary, the problem cannot be formulated as a regression problem any more. Instead, we replace the target regression values by a probability function *f*, as illustrated in figure 3, that is defined over a plane

3.1 $$f(a)=\max_{i}∼\exp\left(-\frac{∥p^{\left(i\right)}-a{∥}^{2}}{2\cdot \rho^{2}}\right),$$

in which $p^{\left(i\right)}$ represents the position vector of the *i*th sphere and *ρ* is a smoothing factor. The vector a is any coordinate for which we want our model to predict the likelihood of a source present.

We thus have two instances of the 2D target probability function, one for each array, for which we define the following discrete domain for array *A*:

3.2 $$D_{A}=\{(x,d_{A})|x\in \{-1.5,\;-1.5+\delta_{x},\;\ldots ,1.5\},d_{A}\in \{0,\delta_{d},\;\ldots ,2\}\}.$$

Figure 3 depicts a discretized example target function for two sources in a single plane. We choose the domain for *x* to be from −1.5 to 1.5 in 32 parts and for *d*_*A*_ from 0 to 2 in 22 parts. This fully encompasses the bounded space of motion for the source (see §3.1.2).

#### 3.1.2. Moving spheres in three dimensions

In order to extend the problem to three dimensions, we consider a planar projection for each spherical source that is defined by the location of sensor array *A* and the $R^{3}$ position vector p of the source.

To conveniently work out the planar projection of source motion with respect to the sensor array, we construct the source’s velocity vector v in $R^{3}$. Next, we project this source velocity v on the plane spanned by the array *A* and the source position p to determine a projected velocity vector $v_{A}$.

We know that the velocity component parallel to the array *A*, assuming the array is aligned with the *x*-axis, is simply $v_{Ax}=v_{x}$. The orthogonal velocity component $v_{Ad}$ requires taking into account both the *y* and *z* components of the position and velocity vector. This orthogonal component is spanned along $p_{yz}={\left[0,p_{y}-A_{y},p_{z}\right]}^{⊤}$ and is thus given by

3.3 $$v_{Ad}=v\cdot \frac{p_{yz}}{∥p_{yz}∥}.$$

The third velocity component that is orthogonal to the projected plane has no contribution to the measured fluid velocity at the sensor array and is therefore neglected in this study.

From the parallel and orthogonal components of the projected velocity vector $v_{A}$, it is trivial to obtain the absolute velocity *W*_*A*_ and angle φ_*A*_. Finally, we find $d_{A}=\sqrt{{\left(p_{y}-A_{y}\right)}^{2}+p_{z}^{2}}$ and substitute the projected parameters in equations (2.1), (2.2) and (2.3), fully defining the 1D velocity pattern for array *A* for any source in a $R^{3}$ volume.

#### 3.1.3. Resolving three-dimensional ambiguity

If we only use a single sensor array, we can estimate the *x*_*A*_ coordinate and distance *d*_*A*_ of a source with respect to array *A*. This evidently causes ambiguous situations where the source could be anywhere in a ring around this array, since the *y* and *z* coordinate of the object are combined in *d*_*A*_.

**Figure 4.**
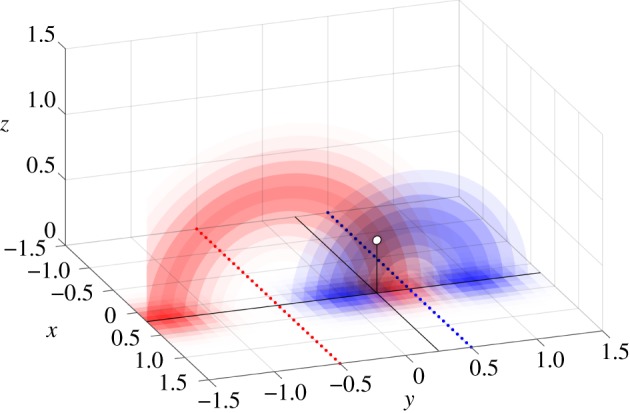
The planar probability grid functions of both arrays are ambiguous in a torus around their array. Shown here is a cross section of the probability tori for a single source. The intersection of maximal probability corresponds to a source’s location in $R^{3}$. (Online version in colour.)

By adding a second array, we have two instances of the probability function, one for each sensor array. Both instances map to toroidal shapes that intersect at the source’s target position, as indicated in figure 4, thereby collapsing the ambiguity. For simplicity and without loss of generality, we will assume that the first array, *A*_1_, is placed at *y* = −0.5 while the second array, *A*_2_, is placed at *y* = 0.5.

### 3.2. Data synthesis

**Figure 5.**
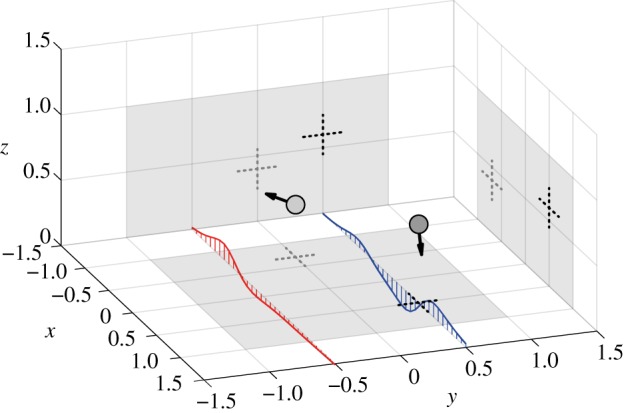
Illustration of a situation with two spheres in a 3D environment together with the velocity patterns of two arrays in the bottom plane aligned with the *x*-axis. The grey backdrops indicate the motion bounds and source location with cross hairs. (Online version in colour.)

We simulate spherical sources moving within a bounded $R^{3}$ space in a basin, generating fluid flow that is measured by the arrays as fluid velocity (m · s^−1^). This bounded space (figure 5) is fully encompassed by the discretized domain *D*_*A*_.

#### 3.2.1. Source motion

For our data synthesis, we consider a simulated environment in which multiple spheres move through a basin *V* measuring 3 × 3 × 1.5 m^3^ (figure 5). A source is allowed to move in a bounded space with *x*, *y* ∈ [−1, 1] and *z* ∈ [0, 1]. Each sphere moves with a constant speed *W* of 0.05 m · s^−1^ and their direction of motion is described by two angles (*ϕ*, *θ*), where *ϕ* is the azimuthal angle in the *x*, *y*-plane and *θ* is the polar angle with respect to the positive *z*-axis. The direction of motion of each sphere is changed according to

3.4 $$\begin{array}{rl} & \;\phi_{t+1}=\phi_{t}+\text{unif}[-1\;\text{rad},1\;\text{rad}]\\ \;\text{and}\; & \;\theta_{t+1}=\theta_{t}+\text{unif}[-1\;\text{rad},1\;\text{rad}]. \end{array}$$

In case the source is about to move outside the bounded space, we let it ricochet as in [21]. Here, the reflected angle is the same as the incident angle.

At any point in time, at least one and at most two spheres can be in the basin. Spheres disappear with a probability of 5% each time step while reappearing at a random location with the same probability. This results in a dataset where one source is present 53% of the time, while in the other cases two sources are present.

#### 3.2.2. Velocity pattern sampling and noise

The velocity patterns are sampled from two simulated sensor arrays placed at the bottom of the basin. We obtain these velocity patterns via the fluid model described in §2.1. For each sensor array, we can simply sum the contributions from each sphere because of the properties of the assumed potential flow.

We then introduce relative noise on top of these sampled velocity patterns, via a noise level parameter *n*. This allows us to make fair comparisons of the network for different signal-to-noise levels. The parameter *n* expresses a ratio of the input’s (i.e. the water velocity) standard deviation, denoted $\sigma_{\forall }$. The computation of this standard deviation considers the velocity patterns for all time steps and all sensors in both arrays. The noise is added by sampling from a normal distribution with mean 0 and a chosen variance of $n\cdot \sigma_{\forall }$.

### 3.3. CNN implementation

**Figure 6.**
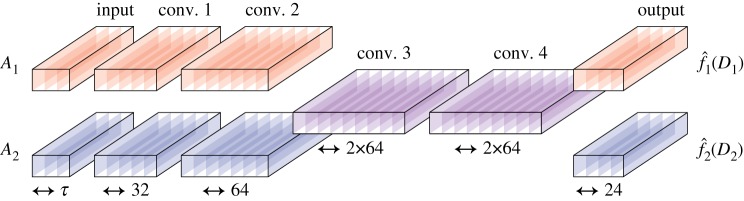
Illustration of the default network architecture. The colour of each layer indicates to which stream it belongs. In this case, the third and fourth layer are merged to allow the two streams to exchange information. (Online version in colour.)

The neural network receives 1D velocity patterns of both sensor arrays and is tasked to approximate two probability grids, one for each array. Table 1 and figure 6 provide an overview of the default CNN architecture, which is further discussed in the following subsections.

**Table 1.** Overview of the default neural network architecture. Where two kernel sizes are listed, each is used for half of the kernels. When the streams are merged, the kernels concatenate so that the total number of kernels per layer is unaffected.

layer description	output merged/split	kernels per stream	kernel size	activation function
input	split	*τ*	—	*x*
conv. 1	split	32	5,7	tanh (*x*)
conv. 2	split	64	5,7	max (0, *x*)
conv. 3	merged	2×64	5,7	max (0, *x*)
conv. 4	merged	2×64	5,7	max (0, *x*)
output layer	—	24	5	sigmoid (*x*)

#### 3.3.1. Input mapping

The magnitude of the sampled velocity patterns can have a considerable dynamic range, given the cubed relation with the distance to the source; see equation (2.3). To normalize and map this dynamic range of the input, we squeeze the activation at the first hidden layer in the range of [−1, 1] by using the hyperbolic tangent function.

As a first input augmentation, we consider feeding both attenuated and amplified versions of the input to the first hidden layer before normalization. The hyperbolic tangent function shows differences in its output more clearly when its input is around 0. Therefore, using different amplification levels simultaneously will allow the network to capture the dynamics at different magnitudes more adequately than a system with a single amplification level.

As an alternative augmentation to the inputs, we also consider an aspect of time-delay neural network architecture that effectively introduces history. In the network architecture, we vary a time-delay frame parameter, *τ*. When *τ* = 1, we only use the current velocity pattern for each array, so no effective history is added. In case of *τ* > 1, the input is constructed via concatenating velocity patterns of previous time steps. It is hypothesized that using past velocity patterns will help the model to interpret otherwise ambiguous situations during testing. Moreover, it might also help to reduce the impact of input noise.

From this point forward, we consider the amount of sensors that sample a velocity pattern as the width of the input, i.e. 32. The additional past velocity patterns that are added as effective history are considered depth slices of the input.

#### 3.3.2. Convolutional streams

The velocity patterns from both arrays are sampled at equidistant locations. This geometry introduces a spatial relation between the shape of the velocity patterns and the position of the source (see §2.1). The convolutional layers in this neural network architecture are especially suited to parse this type of data, since these layers effectively perform 1D convolutions that can make use of this spatial relation.

The convolutional layers are used in combination with the rectified linear unit (ReLU) activation function: *f*(*x*) = max (0, *x*) [32]. The usage of the ReLU activation function reduces the vanishing gradient problem and provides sparsity to the feature representations in the network, which in turn helps for linear separability of features within a layer [32].

To maintain the local spatial relations throughout the convolutional stream, only convolutional layers are used, as opposed to regular CNNs which also incorporate pooling layers. The series of convolutional layers combine to *convolutional stream*s, one for each sensor array. These streams start at the input (index 0) and end at the output layer (index 5). Both streams have the identical task to map an input velocity pattern to its probability grid as the desired output. Hence, we can use the exact same weights for each convolutional stream that predicts one of the two probability functions. In other words, we can share the convolutional streams’ weights. Sharing the weights results in reusing the same kernels in both streams. Disabling sharing allows separate filter kernels to be trained for each convolutional stream.

The convolutional layers all have a width of 32 (just as the input) and a stride of 1. This width is maintained by zero padding the input of each convolution at both ends. The depth of the convolutional layer determines the number of filter kernels.

In addition to weight sharing, we can also *merge* both convolutional streams. This creates a single stream that is responsible for predicting the probability output for both sensor arrays. The streams are merged by concatenating the outputs of hidden layers at a certain layer index, which doubles the layer depth from that point on. In our experiments, we vary this layer index, shifting the merging point. After this merging point, the subsequent layers will receive information from both sensor arrays.

#### 3.3.3. Output mapping

For each sensor array and accompanying convolutional stream, we have one target probability function. At the end of the convolutional stream, two different methods are used and compared to map the activation to the output layer. By only using convolutions, we have maintained local spatial relations; this enables us to use the last convolutional layer directly for this mapping. We also investigate replacing the last (output) layer with a fully connected layer. This type of layer may uncover patterns that stretch over a wider part of the sensor arrays, at the increased risk of overfitting. In both cases, the output layer has a width of 32 and a depth of 24, reflecting the chosen discretization of the domain *D*_*A*_.

Since our target output has values in the range [0, 1], we decided to use a sigmoid function ς(x)=1/(1+exp⁡(−x)) at the output layer, which ensures that the output is in that range.

### 3.4. CNN optimization

This section describes the default CNN settings, how we train the CNN and the chosen architecture variations for these settings.

#### 3.4.1. Optimization criterion

The CNN is trained to estimate two 2D discretized probability functions based on two sampled velocity patterns, one from each array. The network minimizes a custom loss function that takes into account the true and predicted probability as well as a weight regularization term. The exact definition of the loss function that we minimize is

3.5 $$\;\begin{array}{rl} C(A,a) & ={\hat{f}}_{A}(a)\cdot \log f_{A}(a)\\ & \;+(1-{\hat{f}}_{A}(a))\cdot \log(1-f_{A}(a)) \end{array},$$

3.6 $$\;S(A)=\sum_{a\in D_{A}}-C(A,a),$$

3.7 $$\;W(w)=\frac{1}{2}\sum_{i}w_{i}^{2}\;$$

3.8 $$\;\text{and}\;L_{\mathrm{total}}=S(A_{1})+S(A_{2})+\lambda W(w).$$

Equation (3.5) shows the binary cross-entropy loss for a single position a for array *A*, where *f*_*A*_(***a***) is the actual probability and ${\hat{\;f}}_{A}(a)$ is the predicted probability for said position. The binary cross-entropy loss has the desirable property that, when combined with sigmoid activations, its gradients are of approximately the same magnitude across all possible preactivation values.

Equation (3.6) shows the loss for a single array *A*, which simply sums the losses over all positions in the corresponding domain *D*_*A*_.

Equation (3.7) is a regularization term in which w is a vector that contains all trainable parameters of the neural network. This term penalizes large weight vectors, making the whole network less prone to overfitting [33].

Finally, equation (3.8) shows the total loss for a single sample (i.e. a single input and output pair) in which *λ* governs the contribution of the regularization term for the trainable weights. In this study, we use batches of 64 samples for updating the CNN weights.

#### 3.4.2. CNN variations

Our experiments are designed to characterize the influence of design decisions that would be relevant for an ALL system with CNNs. The parameter settings for the default model are listed in table 2. We consider the following variations to the default model.

— *Noise level*. We assess the influence of noise on the final performance of the system and choose *n* = 0.0001, 0.001, 0.01, 0.02.
— *Amplification levels*. We compare a system with a single input amplification level of 1000 with another system that uses the concatenated input of three input amplification levels: 100, 1000 and 10 000. This causes the latter system to have three times as many input depth slices as the former.
— *History length*. We compare different history sizes *τ* = 1, 2, 3, 4 to see whether the system benefits from integrating the inputs of multiple time steps.
— *Merging index*. We vary the merging index from 0, where the input of both arrays is concatenated and parsed by a single stream, to index 5, where no merging takes place.
— *Weight sharing*. For the unmerged layers, we can choose to share the weights by reusing the same kernels in both convolutional streams, or disable sharing so that we use separate kernels for each stream.
— *Output layer*. We compare a network that has a convolutional output layer, forming a fully convolutional neural network (FCNN) with a network that has a fully connected output layer (CNN+FC).

**Table 2.** Default model and hyperparameters.

parameter	symbol	value
first sensor array	*A*_1_	*y* = −0.5
second sensor array	*A*_2_	*y* = 0.5
sphere radius	*r*	0.05
smoothing factor	*ρ*	0.2
relative noise level	*n*	0.001
input range factors		[1000]
history length	*τ*	3
merging index		3
weight sharing		enabled
output layer		FCNN
learning rate	*η*	10 × 10^−3^
regularization coefficient	*λ*	10 × 10^−4^

#### 3.4.3. Validation

We create five different pairs of train and validation data, each generated independently using the model as discussed in §3.2. For each of these pairs, the train data contain 10 000 samples, while the validation data contain 2000 samples.

### 3.5. Location decoding in three dimensions

**Figure 7.**
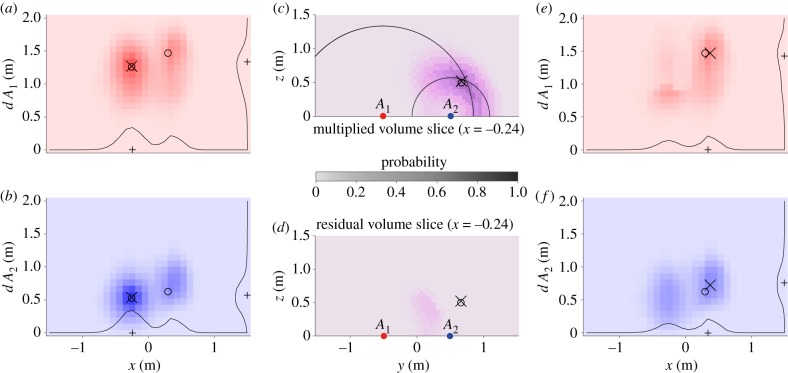
Steps for location decoding of two sources in three dimensions. The circles denote the two real source locations, the crosses show the reconstructed locations, and the pluses indicate discrete maxima for the probability grid. (*a*,*b*) The CNN 2D probability estimation, which is used to locate a first source. (*c*) One slice of a volume, resulting from projecting the probability grids to $R^{3}$ and multiplying the volumes resulting from *A*_1_ and *A*_2_, respectively. (*c*) The arcs correspond with the ‘plus’ symbols for *d* *A*_1_ and *d* *A*_2_. (*d*) A slice of the residual volume after 3D-aware filtering. (*e*,*f*) The residual probability grids, constructed from the residual volume. (Online version in colour.)

Finally, we estimate $R^{3}$ positions $\hat{p}$ from the CNN output. Using the output 2D probability grids, we present a 3D-aware algorithm to detect both the number of sources and their location (figure 7). This algorithm benefits from being 3D-aware, since there could be two distinct objects that have a similar distance to one of the arrays, effectively masking each other in two dimensions. This algorithm is described here in detail using an example; a formal description in the form of pseudocode may be found in the electronic supplementary material.

We first detect the *x*-coordinate of the highest probability by flattening and adding both probability grids and taking the maximum of the resulting probability line (‘+’ in figure 7*a*,*b*). Then, for each array, we find the *d* coordinate with the maximal value for that *x*-coordinate and fit a Gaussian (see equation (3.1)) near the maximal coordinates to yield ($\hat{x}$, $\hat{d}$) coordinate pair estimates *E* (‘×’ in figure 7*a*,*b*). From the estimates $\hat{d_{1}}$, $\hat{d_{2}}$ and the array *y*-coordinates, it is trivial to work out the $\hat{y}$ and $\hat{z}$ estimate for the first source, completing the first position vector.

The next step is to create probability volumes for each reconstructed grid, by effectively rotating these grids, as indicated in figure 4. We choose a voxel size of $\left(\delta_{x},\;\delta_{y},\;\delta_{z})=(\frac{3}{31},\;\frac{3}{63},\;\frac{3}{63}\right)$ and fill each voxel with the value of the nearest mapping to 2D coordinates. We use element-wise multiplication to combine both arrays' probability volumes to a single volume. A slice of this volume is shown in figure 7*c*.

To remove the estimated probability values of the already found source, we calculate two probability grids from $\hat{x}$, $\hat{d_{1}}$ and $\hat{d_{2}}$ using the target function from equation (3.1) and similarly create a probability volume. We then subtract this probability volume from the prior and map the residual volume (figure 7*d*) back to two residual probability grids (figure 7*e*,*f* ). This remapping consists of two steps. First, each pixel in the grid is filled with the maximal value found from the inverse rotational mapping. Then, for each value in the remapped probability grids, we take the square root, since this value originates from a multiplication.

We then repeat the first step of finding ($\hat{x}$, $\hat{d}$) coordinate pair estimates. As is made visible in figure 7*e*,*f*, the 3D filtering method also leaves residual probability at the position of the first source. We, therefore, use two thresholds to determine whether a second detection constitutes a second source. First, the summed probability of the residual probability grids from the last step should be higher than the expected value for target probability grids containing a single source, in this case 55. And second, its estimated position should be further than 0.55 m to the first object's estimated position.

As an error metric for object localization, we report the localization error in MED (m) per object. For samples where we expect two sources, we report how many sources were detected per sample.

## 4. Results

As described in §3.4.2, several types of variations for the CNN architecture were considered, each given a different colour in upcoming figures. We assess both the loss and accuracy with regards to reconstructing the target probability functions, as well as the localization error from the iterative detection algorithm using these reconstructions.

### 4.1. Probability grid reconstruction

In terms of CNN training loss (table 3), the default hyperparameter settings seem to produce the best performing network in terms of 2D probability reconstruction. The fact that the loss values are relatively large is a result of summing the binary cross entropy per location in equation (3.6), rather than taking the average.

**Table 3.** Target probability reconstruction metrics. The default setting entry (D) is repeated where appropriate. Bold indicates settings in which the MSE is significantly lower than the default setting.

	loss	MSE	±s.d.
default	142.3	5.28 × 10^−3^	5.99 × 10^−4^
*τ* = 1	143.0	**4.83 × 10^−3^**	3.71 × 10^−4^
*τ* = 2	143.5	5.25 × 10^−3^	4.90 × 10^−4^
*τ* = 3 (D)	142.3	5.28 × 10^−3^	5.99 × 10^−4^
*τ* = 4	143.8	5.33 × 10^−3^	6.57 × 10^−4^
$0.0001\sigma_{\forall }$	142.4	**4.84 × 10^−3^**	3.80 × 10^−4^
$0.001\sigma_{\forall }$ (D)	142.3	5.28 × 10^−3^	5.99 × 10^−4^
$0.01\sigma_{\forall }$	147.6	6.07 × 10^−3^	6.83 × 10^−4^
$0.02\sigma_{\forall }$	148.5	6.35 × 10^−3^	6.91 × 10^−4^
merge at 0	153.3	7.03 × 10^−3^	6.20 × 10^−4^
merge at 1	151.3	6.86 × 10^−3^	8.53 × 10^−4^
merge at 2	148.4	6.46 × 10^−3^	8.29 × 10^−4^
merge at 3 (D)	142.3	5.28 × 10^−3^	5.99 × 10^−4^
merge at 4	146.0	5.58 × 10^−3^	5.36 × 10^−4^
no merging	156.3	7.57 × 10^−3^	8.42 × 10^−4^
multi-range	143.7	**4.35 × 10^−3^**	1.99 × 10^−4^
no sharing	143.4	5.41 × 10^−3^	7.09 × 10^−4^
fully connected	166.9	8.40 × 10^−3^	3.29 × 10^−4^

An example of the CNN output for two sources can be seen in figure 7*a*,*b*. Here, and in most other samples, the reconstructed probability grids do not show perfect Gaussians, but the maxima in these grids often coincide with the actual position of that object.

There are four variations that outperform the default settings in terms of the average probability reconstruction MSE. The first two variations (*τ* = 1 and *τ* = 2) have a shorter history length. Given the standard deviations, the effect of this parameter on the reconstruction quality is marginal and provides no significant improvement. The third variation to outperform the default settings is the lowest relative input noise setting (0.0001*σ*_∀_). This is expected, as the emulated sensors effectively have a higher sensitivity. The final outperforming variation (multi-range) concatenates amplified and attenuated versions of the input for the CNN input. While the training loss is slightly higher than default, this variation performs significantly better in terms of reconstruction quality.

### 4.2. Three-dimensional position reconstruction

Using the reconstructed 2D probability grids from the CNN, we used the iterative algorithm explained in §3.5 to detect sources and determine their position in three dimensions.

Figure 7 shows the process for a single sample for determining the position of two objects. It shows the advantage of taking our approach for detecting maxima in these 2D maps.

The first estimates for simply picking the maximum pixel (indicated with pluses) is a good start, but only provides estimates from a discretized coordinate system. As is more clearly visible in figure 7*c* (the intersection of the two arcs), this initial estimate tends to overestimate the distance. While the final position from the fitting procedure does not necessarily coincide with the ground truth source positions, these final estimates do clearly reflect the maxima of the probability grids.

**Figure 8.**
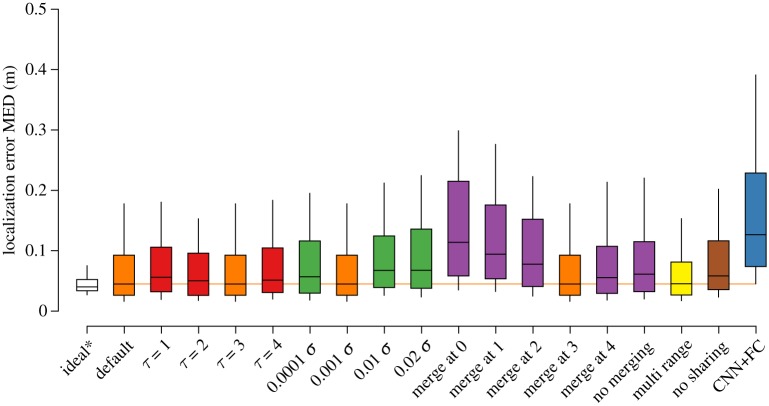
Localization error for samples containing a single source. The whiskers and boxes mark the 10%, 25%, 50%, 75% and 90% percentiles of the error distribution. The performance of the default model is repeated where appropriate. All sources were found in this subset of the dataset. The ideal* case reflects the performance on a hypothetical perfect probability grid reconstruction. (Online version in colour.)

**Figure 9.**
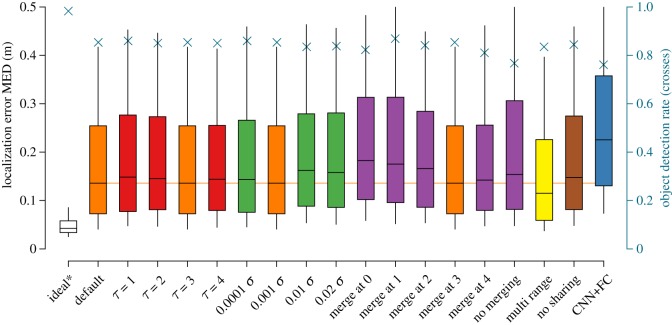
Average localization error for samples containing a double source. The whiskers and boxes mark the 10%, 25%, 50%, 75% and 90% percentiles of the error distribution for the left axis. The right axis and crosses denote the object detection rate, where 1 indicates that both sources are found and 0 indicates that none are found. The ideal* case reflects the performance on a hypothetical perfect probability grid reconstruction. (Online version in colour.)

Figures 8 and 9 show the localization error distribution for a hypothetical perfect probability reconstruction and the reconstruction from each type of CNN model alteration. The performance of these model alterations, broadly speaking, aligns with the quality of probability grid reconstruction.

For detecting the location of a single source (figure 8), the default model has the lowest median error, but the shorter tail of the ‘multi-range’ model suggests that its performance is more consistent. All models perform reasonably well, given that the average random prediction error in a bounded volume of 2 × 2 × 1 m is 1.132 m. Especially, the default model and the ‘multi-range’ model result in a localization performance that is close to optimal.

In the cases where two sources are present (figure 9), the performance of the localization algorithm is only slightly affected in the ideal case. For the CNN probability grid reconstructions, the differences between most CNN models decrease, while the localization error per source more than doubles overall. With respect to the model alterations, most trends visible in the case with one object are also visible here, with one notable exception. Here, the ‘multi-range’ model outperforms the default model, signified by its lower median and its shorter third quartile.

In some cases, the pipeline described in §3.5 was not able to reliably determine a second source. The default CNN model has an object detection rate of 85.4%. Only the *τ* = 1, ‘merge at 1’ and ‘multi-range’ variants have a detection rate that is on par.

## 5. Discussion

Here, we discuss the overall results, followed by the effects of the extensive range of architectural design variations for the CNN, as well as the effectiveness of the incorporated localization method. This is followed by a discussion on the detection limits of the approach and directions for future research.

### 5.1. The processing pipeline

The final average error in localization performance can be thought of as a cascading effect of imperfect maxima localization, which is influenced by an imperfect probability reconstruction, which can be caused by unbalanced sampling in the simulation or a suboptimally configured CNN. Uncertainty or information loss can be introduced and interact at each of these stages.

While better probability reconstructions tend to result in better localization performance overall, optimizing these intermediate steps does not guarantee optimizing the end result. This is made apparent by the reconstruction performance of a CNN with *τ* = 1, which is the best variant in terms of MSE. It is however outperformed in localization by a CNN with *τ* = 3. In this case, the values in the reconstructed maps of the CNN with *τ* = 1 are closer to the ground truth on average, but their maxima may have been further away from the actual position than those in the case with *τ* = 3.

#### 5.1.1. CNN design variations

There are a number of parameters that were not optimized in detail, such as the learning rate, the regularization loss, the number of hidden layers and the number of neurons per hidden layer. It is likely that our system can be improved by performing a grid search, random search or evolutionary search to optimize these hyperparameters [34,35]. We discuss here the effects of the parameters that were varied.

*History *τ*.* First of all, the history length indicated by the *τ* parameter seems to have a marginal effect on the reconstruction performance. Perhaps surprisingly, a history length of *τ* = 1 results in the most accurate model. This could be explained by the fact that it is easier to generalize from single time steps than it is to generalize from multiple time steps.

Perhaps in a more complex setting it might still be valuable to incorporate previous time frames, as has been shown for localizing a single source using regression [24]. One such situation that may benefit from taking history into account is when objects turn more slowly than the current maximal 1 rad (57 degrees) per second or when the objects can vary their speed. These more complex situations would likely reduce the performance of the system as currently optimized. It would require more training data to be generated for tuning any inverse method for localizing these objects.

*Noise.* There seems to be no obvious relation between the noise level and localization performance. We see that, when the noise level is 10 times lower than the default, we obtain a marginally worse-performing model. The performance with higher noise levels follows intuition and also degrades. It is likely that some form of noise may have helped to prevent overfitting for the default CNN architecture.

*Merging convolutional streams.* Similarly, the default CNN model may have the optimal merging index. We note that the number of neurons is kept the same for any merging index, and thus should not cause a difference in the performance.

Merging at the input (index 0) leads to the worst average localization performance. In this case, the streams are not separated at all. As we postpone the merging point, we initially observe a gradual improvement. However, after merging index 3, we observe impeded performance. Perhaps this could be explained by the fact that the earlier layers will be able to extract the local characteristics of the velocity patterns of a single sensor better when not affected by the velocity patterns of the other array. This makes it easier for the first few layers to generalize the representation that they learn, as the number of possible inputs per layer is greatly reduced by splitting the two streams.

In the case of merging index 4, there is no hidden layer between the merged streams and the output. This prevents the CNN from making use of nonlinear processing, which is likely to be beneficial. This might also explain why we see a relatively poor performance when no merging is used at all.

*Amplification levels.* Using multiple amplification levels results in a considerable improvement compared with the default model for probability function estimation and localization, especially when two sources are considered.

The cubic term in equation (2.3) causes the input to be in a large dynamic range. By using multiple amplification levels, the network can encode salient differences in sensor inputs at multiple scales, which improves the overall performance. This effect is more prominent in samples containing two objects. In these cases, both near (strong) and far (weak) sources are likely to be processed in a favourable dynamic range in one of these amplification levels.

*Weight sharing.* Disabling weight sharing between the streams seems to have a negligible effect on performance. Perhaps if more arrays were aligned, weight sharing may be more beneficial, since it makes training more effective.

*Adding a fully connected layer.* It seems that using a fully connected layer at the back of the network, instead of a final convolution layer, severely impedes performance. Since the amount of trainable weights is increased considerably, it is likely that this is a result of overfitting. Perhaps with a bigger dataset, the CNN+FC may still prove to be useful.

#### 5.1.2. Iterative source detection

The 3D-aware filtering method provides an iterative approach to detecting multiple objects, as detected by multiple arrays. A consequence of the 3D-aware filtering method is that, after removing the most prominent source, there is some residual probability left on that location (figure 7*d*). In our case, via reasonably chosen thresholds, the expected summed probability for a single source and a minimal distance, we were able to detect a second object in most cases. There are two main advantages of this method.

First, objects which are masked in two dimensions, can still be detected. It could happen that two objects are positioned at (0, 1, 1) and (0, 0, 1), respectively. These two sources would have an identical distance to *A*_2_, and thus sensed as a single object by that array, but they can be discerned and detected as separate objects via array *A*_1_.

Secondly, without specifying how many sources should be detected, this algorithm can detect an arbitrary number of sources. As it is the first demonstration of localizing an arbitrary number of objects with an ALL, we cannot readily compare this method with other (regression) inverse methods in the literature without adjusting them.

We note that the current data generation does allow for two sources to be spawned at the exact same place, with no minimal distance enforced. This has in some cases led to a single perceived object, which affected the detection rate. For future simulations and experiments, a minimal distance between objects may improve the quality of probability reconstruction and the source detection performance.

### 5.2. Detection limits and future research

Hydrodynamic imaging is a near-field modality. The distance range in which this near-field localization method can be used is limited. The hydrodynamic information carried through the water deteriorates with the cube of the distance *d*^3^, while for (far-field) sound signals this is a factor of the distance squared *d*^2^, which is the case for sonar. While the detection range for sonic detection is bigger, hydrodynamic detection can be more effective in the near-field.

Scaling up this principle is therefore not trivial, as hydrodynamic imaging is mostly beneficial in the near-field range. This near-field can be extended by increasing the source’s speed and volume. We could, for instance, scale up the source properties, set-up and domain with a constant factor. However, even when the hydrodynamic signals can be amplified to counter the effect of an increased distance, the resolution of sampling a velocity pattern will drop and likely impair the system. At further distances, sonar-based solutions have a clear advantage.

The application of hydrodynamic detection of objects can therefore not scale up indefinitely, but the range can be extended beyond the current chosen domain. It has been shown that the source can be positioned in an area next to the array and still be detected reliably using different types of artificial neural networks [14,20,24]. It is, therefore, not necessary for the whole velocity pattern to be sampled within the area directly in front of the array; the domain can extend beyond the length of the array. The CNN might therefore be less effective in situations where only a part of the velocity pattern is detected, since some of the spatial characteristics are not sampled.

The current iterative method of localization performs equally well in situations of one and two objects present, and is therefore not a bottleneck in this pipeline. However, even in the ideal case where the method is presented with a perfect probability reconstruction, some errors remain. The lower bound on this error might be a result of the unbiased sampling of locations; in some cases, two objects were instantiated in nearly the same location.

For future research, it should therefore also be investigated whether the current iterative position reconstruction method is the most appropriate in cases with non-overlapping or even more objects. Other peak-detecting algorithms may be equally able to cope with the 2D masking in probability grids constructed from 1D velocity patterns resulting from objects moving in 3D space.

Our research can be extended by further increasing the variation on the input. One could increase the number of simultaneous objects and allow different sizes, different shapes and a variable speed. It is evident that the more variations the system has to learn to cope with, the more data will be required to train the system. The results obtained in this research suggest that there is potential for our implementation to address such complicated scenarios.

## 6. Conclusion

In this paper, we assessed a new approach to underwater object localization using a simulated ALL with CNNs. Via the iterative 3D-aware position estimation algorithm, we have shown that multiple spheres can be detected simultaneously in a 3D space by using two sensor arrays placed in parallel.

The most significant CNN model improvement is a result of providing the input at several amplification levels. Since the input was compressed and normalized between −1 and 1, these additional amplification levels effectively made the CNN more compatible with the considerable dynamic range of the input. Additionally, by placing two ALLs in a parallel configuration, the two convolutional streams of the CNN could be merged and exchange information, which is beneficial for localization performance.

We have, therefore, demonstrated that the combined system is suitable for localizing multiple moving objects in a bounded volume.

## Supplementary Material

Pseudocode for iterative 3D-aware filtering and localisation

## Data accessibility

The iterative localization algorithm is described using pseudocode in the electronic supplementary material; an implementation of the algorithm and the ‘default CNN’ heat map output can be accessed via https://doi.org/10.17605/OSF.IO/V5ABF.
