# Supplementary material for: Three-dimensional multi-source localization of underwater objects using convolutional neural networks for artificial lateral lines
Source: J R Soc Interface. 2020 Jan 22;17(162):20190616. doi: 10.1098/rsif.2019.0616 (PMC7014811; doi:10.1098/rsif.2019.0616)
Supplement: Pseudocode for iterative 3D-aware filtering and localisation [file rsif20190616supp1.pdf]

Pseudocode iterative 3D-aware localisation algorithm

This supplementary material is part of the paper "3D Multi Source Localisation of Underwater Objects using Convolutional Neural Networks for Artificial Lateral Lines", by Ben J. Wolf, Jos van de Wolfshaar, and Sietse M. van Netten, published in the Royal Society Interface.

The iterative 3D-aware localisation algorithm, as explained in section 3.5 of the paper, is described here with pseudocode. This description indicates the overall structure of the iterations in algorithm 1. The three non-trivial sublevel algorithms are also described in algorithms 2, 3, and 4 respectively.

Data: probability grids $f_1(D_1)$ and $f_2(D_2)$.
Result: position vector $\hat{\boldsymbol{p}}$ per detected source.

$H_1 \longleftarrow f_1(D_1)$
$H_2 \longleftarrow f_2(D_2)$
do
  return a location
  $\hat{x}, \hat{y}, \hat{z} \longleftarrow \texttt{findSource}(H_1, H_2)$
  $\hat{\boldsymbol{p}} \longleftarrow \hat{x}, \hat{y}, \hat{z}$
  filter found source in 3D
  $V_{\text{cur}} \longleftarrow \texttt{map2volume}(H_1, H_2)$
  $H_{\text{est1}}, H_{\text{est2}} \longleftarrow \texttt{2Dgauss}(\hat{x}, \hat{y}, \hat{z})$
  $V_{\text{est}} \longleftarrow \texttt{map2volume}(H_{\text{est1}}, H_{\text{est2}})$
  $V_{\text{res}} \longleftarrow V_{\text{cur}} - V_{\text{est}}$
  $H_1, H_2 \longleftarrow \texttt{volume2maps}(V_{\text{res}})$
  determine stopping criterion
  totEnergy $\longleftarrow \Sigma H_1 + \Sigma H_2$
while *totEnergy* ≥ 55

Algorithm 1: iterative localisation algorithm

Input : 3D volume V
Output: 2D maps H_1, H_2

$H_1, H_2 \longleftarrow \texttt{zeroes}$
for *every coordinate* $\boldsymbol{c} \in$ *volume* V **do**
  determine distance to each array
  $d_1 \longleftarrow \sqrt{\boldsymbol{c}_z{}^2 + (\boldsymbol{c}_y + 0.5)^2}$
  $d_2 \longleftarrow \sqrt{\boldsymbol{c}_z{}^2 + (\boldsymbol{c}_y - 0.5)^2}$
  find nearest 2D coordinates to write
  $H_1(\langle \boldsymbol{c}_x \rangle, \langle d_1 \rangle) \longleftarrow \texttt{maxVal}(H_1(\langle \boldsymbol{c}_x \rangle, \langle d_1 \rangle), V(\boldsymbol{c}))$
  $H_2(\langle \boldsymbol{c}_x \rangle, \langle d_2 \rangle) \longleftarrow \texttt{maxVal}(H_2(\langle \boldsymbol{c}_x \rangle, \langle d_2 \rangle), V(\boldsymbol{c}))$
  return element-wise square rooted maps H_1, H_2
  $H_1 \longleftarrow \sqrt{H_1}$
  $H_2 \longleftarrow \sqrt{H_2}$
end

Algorithm 2: volume2maps

Input : 2D maps H_1, H_2
Output: position estimate $\hat{x}, \hat{y}, \hat{z}$

find coordinates of maximum value
$E_x \longleftarrow \texttt{maxCoord}(\Sigma_x(H_1) + \Sigma_x(H_2))$
$E_{d1} \longleftarrow \texttt{maxCoord}(H_{1,x=E_x})$
$E_{d2} \longleftarrow \texttt{maxCoord}(H_{2,x=E_x})$
fit equation (6) near initial estimate position
$\hat{x_1}, \hat{d_1} \longleftarrow \texttt{fit2Dgauss}(H_1, E_x, E_{d1})$
$\hat{x_2}, \hat{d_2} \longleftarrow \texttt{fit2Dgauss}(H_2, E_x, E_{d2})$
transform x, d estimate to R3 position
$\hat{x} \longleftarrow \texttt{mean}(\hat{x_1}, \hat{x_2})$
$\hat{y}, \hat{z} \longleftarrow \texttt{transform}(\hat{d_1}, \hat{d_2})$

Algorithm 3: findSource

Input : 2D maps H_1, H_2
Output: 3D volume V

for *every coordinate* $\boldsymbol{c} \in$ *volume* V **do**
  determine distance to each array
  $d_1 \longleftarrow \sqrt{\boldsymbol{c}_z{}^2 + (\boldsymbol{c}_y + 0.5)^2}$
  $d_2 \longleftarrow \sqrt{\boldsymbol{c}_z{}^2 + (\boldsymbol{c}_y - 0.5)^2}$
  find map values on nearest 2D coordinate
  $V_1(\boldsymbol{c}) \longleftarrow H_1(\langle \boldsymbol{c}_x \rangle, \langle d_1 \rangle)$
  $V_2(\boldsymbol{c}) \longleftarrow H_2(\langle \boldsymbol{c}_x \rangle, \langle d_2 \rangle)$
  return element-wise multiplied volume V
  $V \longleftarrow V_1 \odot V_2$
end

Algorithm 4: map2volume
